# Transactions of the Medical and Physical Society of Calcutta

**Published:** 1834-01-01

**Authors:** 


*Transactions of the Medical and Physical Society of Calcutta.* Volume the Sixth.—Calcutta, 1833. 8vo. pp. 509.

In tracing the progress of civilization through the pages of history, we are continually reminded of the maxim of Bacon, that knowledge is power: we see the countless hosts of Gaul or Thrace yielding to a few disciplined legions; and, in after ages, the humble embassies of distant nations confessing that their only hope was in submission, for that force is rendered irresistible by science. Yet the course of modern events is more gratifying still: they shew us that intellect is not only the parent of power, but the sister of benevolence; and, if the victorious arms of Europe have commenced by destroying, they have ended by renovating. It is thus satisfactory to see the light of science reflected upon those shores whence originally it emanated; to see medicine, the first and greatest of the arts, restored to that antique and hallowed birthplace whence the Father of Physic himself drew his inspirations; so that, if the Western sceptre has sometimes sorely bruised its dusky subjects, yet, like the fabled arrows of the old mythology, it can cure the wounds which it inflicts. These reflections have naturally passed through our minds in perusing the present volume, where we find a native of Madagascar relieved of a fearful disease by the instruments of Civiale, and the bronchocele of Nipâl disappearing before the brilliant discovery of a French chemist.

The following are some of the more interesting and most practical points contained in these "Transactions."

Mr. RALEIGH has a paper on a *Modification of the Oriental Operation of Couching*. He thus details the manner in which the native operators set to work:

"It will be remembered that the instruments employed are a blunt lancet, guarded by thread wound round the blade, and a brass depressing instrument, about four inches long, the extremity of which to be introduced into the eye, forms a solid triangle, having three flat sides, and being about the size of the quill of a common fowl; a puncture being made by the former, through the tunics, the depressor is introduced, for about an inch, behind the lens, the handle of the instrument is then allowed to hang down, and in this position acts as a lever, the lower edge of the puncture forming the fulcrum, whilst the point of the instrument presses against the delicate retina, at the roof of the eye. After remaining a short time in this situation, whilst some invocation is going through, the attempt is made to carry the instrument over the upper edge of the lens, but, from its bulk, instead of accomplishing this object, it frequently happens that the lens is protruded against the iris, and as the shape of the instrument does not admit of fixing the lens, no command over, or power of directing its course, is maintained by the operator; the consequence is, that in the act of depressing, the lens continues to be pushed forward against the iris, from the posterior surface of which the uvea is scraped, and rolling beneath the flat surface of the instrument, its upper edge, and indeed sometimes the whole lens, is forced through the pupil into the anterior chamber. In endeavouring to prosecute the operation, under such unfortunate circumstances, I have on more than one occasion seen the iris torn to pieces, and the chambers filled with blood. The violence which must evidently be inflicted on the retina, ciliary processes, iris, &c. even where the operation is as neatly performed as the clumsy native instruments admit of, is quite sufficient explanation of the fact, of more acute inflammation following this, than the operation as performed by European surgeons." (P. 138.)

To us, accustomed as we are to the delicate touches of European oculists, this would seem more like the first attempt of a butcher to couch with a skewer, than the work of a master in surgery; yet this rude method succeeds so often, that the late Dr. Breton estimated the failures at only ten per cent.: Mr. Raleigh, however, thinks that forty or fifty per cent. is nearer the mark. Moreover, when our Hindoo brethren *do* succeed, they succeed to admiration; and, though they may fail in two points of the triad of medical perfection, they certainly hit the third: if they do not cure *tutò* and *jucundè,* at least they do so *celeriter,*—their operation requires no repetition.

"The reason of this is obvious. By the native instrument, the capsule is separated from its attachments, and carried down entire, together with the enclosed lens, whilst, in performing the operation in the European way, with Hey's, Scarpa's, or other needle, in cases of hard lenticular cataract, it frequently happens, that instead of carrying down the capsule entire, the lens is wrested through it, leaving numerous obtruding shreds, for the removal of which one or more subsequent applications of the instrument are necessary: but more especially in those cases where the cataract is fluid, soft, or only of moderate consistence, the needle ruptures the bag, allows the fluid to escape, or breaks up the substance, without directly removing the opaque body from the axis of vision, and nature is depended on to accomplish the cure by solution, thus in time effecting what may perhaps be considered (as where extraction is resorted to) the most perfect result of the operations for cataract,—the complete removal of the diseased substance from the eye. But to the impatient, and particularly to the natives of India, who anticipate immediate benefit, the repetition of operations, and consequent delay, is by no means satisfactory, nor indeed could it under any circumstances be considered advisable, were we in possession of means for accomplishing the object at once with safety." (P. 140.)

Influenced by these considerations, Mr. Raleigh uses an instrument partly modelled on the native one, and in the following manner:

"In the construction of the instrument, I have endeavoured, as much as possible, to avoid any increase of bulk, beyond that of the common couching-needle, whilst a main object has been, to make it of such form, as to enable the operator to command, and direct the movements of the lens, during the act of depressing. The blade of the instrument (fixed into a handle similar to that of a couching-needle) is about an inch in length; for two thirds of an inch from the handle, the shaft is round, but from the commencement of the extreme third, the blade is gradually dilated into a long oval shape; the solid centre of which portion being cut out, it forms an oval ring, and as the instrument in approaching the extremity makes a gentle curve, and the inner edges of the ring are sloped away, it may be said to form a shallow spoon-shaped blade; at this, which is the widest portion, it measures about the tenth of an inch, but as the thickness does not exceed that of a couching-needle, it passes through the eye in an equally small space.

"It must ever be considered indispensable that the oculist should be competent to perform all the different operations for cataract, and that he should in each case pursue that course which holds out the greatest prospect of benefit to the patient; for in diseases of the eye, as well as in every other branch of surgery or medicine, no one method is applicable to every condition. In order, therefore, that it may not be misunderstood, that I consider the modified native operation worthy of superseding, in all cases, the European mode, I will briefly remark, that it is only in cases where from the appearance of the cataract we may be led to the supposition that the capsule contains fluid, that the lens is too soft to be depressed by the European needle, or that the capsule is too tough to be carried down entire with the lens, that the method now under consideration may be practised with advantage, over the usual European style of operating. The only form of cataract, however, to which the modified native operation is inapplicable, is capsular; in this instance, I conclude, it would be worse than useless to have recourse to it. The manner of performing the operation is as follows. With a lancet-shaped or Beer's knife, a puncture, the eighth of an inch in length, is made through the coats of the eye, about three lines posterior to the junction of the cornea and sclerotic, and sufficiently low down to avoid the central artery; any trifling effusion of blood, &c. being wiped away, the depressor is introduced (its flat surfaces directed anteriorly and posteriorly) behind the lens, and carried as high as its upper edge, when the instrument is turned with its thin edges to face before and behind, and its concave surface downwards, and cautiously insinuated over the margin of the crystalline, which being received within the concavity of the blade of the instrument, is to be gently and gradually depressed; the instrument being at the commencement of this action slightly inclined forwards towards the iris, so as to tilt up the under edge of the lens, which having been carried below the pupil, and so far back as to insure complete separation from its attachments, the handle of the instrument may be rolled between the finger and thumb, to free it from the lens, and withdrawn.

"The principal point of difference between the European and native operation, is, the position chosen for applying the instrument to act on the lens; in the former, the needle passed before the crystalline is applied to the upper and fore part of its body, not on its upper circumference, hence the frequent rupture of the capsule, and wrenching of the lens through it, in the act of depressing. In the latter, the instrument is made to surmount the capsule and lens, and carries them down together without rupturing the former." (P. 141.)

MR. BRAMLEY gives some account of the *Bronchocele of Nipâl.* In three villages, from which he has returns, one third of the inhabitants are affected with this disease. He divides bronchocele into three kinds,—the cellular, the pulsating, and the glandular or lymphatic. He finds *pressure* so useful, that it has sometimes been sufficient alone to accomplish a cure.

CASE I. Bhurrsurie, æt. twenty-six, affected with a pulsating enlargement of the whole of the thyroid gland, was cured in twenty-seven days by wearing a large neckcloth, and rubbing the diseased part with lard.

CASE II. Churù, æt. thirty, was nearly cured by the same treatment.

Four other cases, in which this plan was employed with little advantage, were cured by the iodine ointment. In one case narrated by our author, the use of the Tr. Iodines reduced the testes to one third of their proper size, annihilating at the same time the passion which depends upon them. By discontinuing the remedy, however, these mortifying defects began to abate; but the patient absented himself, and Mr. Bramley could not learn the result.

Our author treated on the whole 116 cases; of these there were

57 perfectly cured,
14 nearly cured,
34 much relieved,
6 slightly relieved,
5 not benefited.

116

Mr. Bramley's tincture is weaker than that generally used in Europe, as it consists only of ten grains of iodine to an ounce of spirit. The dose was from ten to thirty drops once a day. Our author's ointment was at first composed of two scruples of iodine to an ounce of simple ointment, but the quantity was subsequently doubled. Mr. Bramley's success is no doubt partly attributable to the medicine having always been administered, and the ointment used, in his presence.

MR. STEART cures *Cholera* with *Liquor Ammoniæ;* the dose being thirty drops in three and a half drachms of distilled water, to be repeated every five, ten, or fifteen minutes. He also bleeds; and he gives a mild purgative on the morning following the reaction produced by the ammonia. If we understand the abstract given at page 381, it would appear that Mr. Steart lost only one patient out of 124; a degree of success beyond all precedent.

This paper is backed by an appendix written by Mr. Ludlow, the superintending surgeon of the western division, who "came to the conclusion that his practice was unusually successful," and who thinks it his duty "to suggest such practice, and to order a supply of the liquor ammoniæ puræ to all the stations of the division, out of the twenty pounds which has just reached the depôt."

Mr. Twining, in a short and well-written paper, has a few observations *on some of the Effects of Iodine.* It produced pain of the right side in five out of twenty-three cases in which he administered it; and Mr. Twining therefore considers it a dangerous remedy in hepatic affections. He observes also, that Christison refers to two cases in which hepatitis was produced by large doses of iodine.

Mr. Preston writes *on Ligature of the Carotid Arteries in Epilepsy, and some other Diseases.*

Case i. Francis Fullinfan, a robust man, æt. twenty-four, suffering from constant headach and partial paralysis, was admitted into the hospital August 10th, 1831. The right common carotid was tied on the 2d of September, and the left on the 10th of October. "He was discharged on the 11th of November. Vision continued imperfect, but in every other respect he had entirely recovered his health." (P. 396.)

In the next case, which was a combination of epilepsy and hemiplegia, both carotids were likewise tied; but, though the patient was relieved, he was very far from cured.

We cannot commend Mr. Preston for his readiness in performing so very serious an operation when not compelled by absolute necessity; and moreover it has been justly observed by a writer in the "Archives Génerales," that we should be in danger of tying the carotids when the epilepsy depends on organic disease of the brain.

A case of *Traumatic Tetanus,* narrated by Dr. Murray, which was cured by dividing the posterior tibial nerve, is so interesting, that we shall present our readers with a large portion of it.

"The following case of traumatic tetanus occurred on board the ship *James Patterson,* in which I was a passenger from England to Calcutta, and may be deemed worthy the notice of the Medical and Physical Society. The patient, Mr. W. Pyle, a midshipman, aged fifteen, trod on a rusty nail, and received a punctured wound of the left foot between the metatarsal bones of the great toe and adjoining one, on the evening of the 14th August, 1832, at nine p.m. This officer kept his watch during the night, which was rainy and cold, and he suffered much pain in the wound. Thirteen hours after the infliction of the wound I was consulted on this case by Mr. Leslie, the surgeon of the ship, and learned from him that when he first saw the patient, at eight A. M., he complained of a stiffness about his jaws and throat, which had increased very much since that time. His countenance was anxious, and his lips appeared swollen and rather livid. A poultice to the wound was all the treatment that had been employed. On consultation, the following draught was agreed to be given: ℞. Pulv. Camp. ʒss. Tinct. Opii, minim. lxx. Syrupi Simp. ℥j misce, statim sumend. As the jaws were nearly closed, and great difficulty found to get them opened to the extent of a quarter of an inch, a piece of wood of that thickness was inserted between the teeth.

"Half an hour after taking the draught he was visited again; but no beneficial effect had resulted from the opiate. The tetanic symptoms were rather increased, the spasms had partially extended to the muscles of the neck, and the piece of wood was deeply indented by the teeth. The countenance was extremely anxious, and covered with a cold perspiration. The limb was cold, and he said 'it was dead, and that he had little power of moving it.' His pulse was 120, and what may be called irritable, and his situation altogether seemed to be one of great danger. He was ordered to be carried to one of the best cabins, and, after considering all the different modes of treatment that have been usually employed and recommended in tetanus, and finding how indecidedly they were all spoken of, as to affording hopes of cure, I proposed to Mr. Leslie the division of the posterior tibial nerve, (by which the injured part was supplied,) as a remedy that held out a good prospect of success, from its effectually cutting off the communication between the source of irritation and the brain, at the same time that it was an operation easily performed, and unattended with danger or deformity. I proposed also that the original wound should be dilated and cauterized. My proposals being agreed to, so soon as the necessary preparations could be made, the operation was performed. A straight incision, an inch and half in length, was made through the integuments and the aponeurotic fascia, about an inch posteriorly to the malleolus internus, which laid bare the sheath of the vessels; and on dissecting deeper, I easily found the nerve in its usual position. By an aneurism needle, I separated and raised it, so that I might divide it with greater facility and expedition. When thus brought into view, it appeared to us so remarkably large, (being nearly twice the usual size,) though of the natural colour, that a doubt was expressed by Mr. L. about its really being the nerve. To satisfy him on this point, I requested the patient to extend his foot, which he did with difficulty, and in an imperfect manner, as he said he had lost the power of moving it; but he did it sufficiently to shew the difference between the nerve and a tendon, as it did not become tense in this action. The nerve was then rapidly divided by a single cut of the scalpel, which gave great pain, and although he could not articulate distinctly before, on account of the closed state of his jaws, *he immediately opened his mouth with an exclamation.* On looking at his countenance, I was astonished at the striking change in it for the better. I asked him how he felt, and he said 'he was already much better, and that his leg had come to life again,' and expressed at the same time a great inclination to go to stool. There was scarcely any hemorrhage from the wound, which was then simply dressed by bringing the edges of it together by adhesive plaster, with lint and a bandage over it. We then dilated the original wound made by the rusty nail, which also (rather unexpectedly) gave acute pain; but we did not cauterize it, as proposed, the symptoms having already yielded. A poultice, sprinkled with laudanum, was applied over it. His bowels were copiously moved, and, on being placed in bed, he fell into a sound sleep, (without any additional opiate,) which continued without interruption for four hours, and awoke very much relieved from all his former disagreeable sensations. His jaws still, however, felt rather stiff, but, on strong exertion, he could open them nearly to the full extent. The excessive pain in the original wound, of which he had complained previously to the operation, had entirely ceased, and the motion of the limb was now quite restored. It was found, on examination, that the heel and sole of the foot were quite benumbed, but the sense of feeling in the upper part was not affected. At night he got the following powder: Pulv. Opii, gr. ij.; Pulv. Camphoræ, ℈j." (P. 410.)

A bleeding of twelve ounces, which produced syncope, and a number of opiates, completed the cure, which was perfect in every respect, excepting that an entire want of sensation in the little toe and heel remained even after the lapse of six months.

Two cases in which *Lithotrity* was successfully employed are detailed by Dr. Casanova, who is probably the first that has performed this operation in Bengal; and numerous cases of *Lithotomy* are communicated by other surgeons; their chief interest consisting in being practical contradictions of a theory which long bore sway on very slender evidence, namely, that calculus is almost unknown in India.

Mr. Twining has a short paper on *the Comparative Strength of a dark and light coloured Variety of Croton Oil.* The former was the most powerful, "in the ratio of eleven to six; the average purgative effects of seventeen doses of the former being to produce eleven stools, and in that number nine patients were griped, ten complained of a disagreeable sensation of heat in the belly, and two vomited. The average effects of eighteen doses of the light-coloured oil (No. 2,) was [sic] to produce six stools; and, in those patients, seven complained of a sensation of heat in the belly, nine were griped, and one vomited." In two cases, one minim of the stronger oil produced twenty-four stools.

We have thus given an abstract of the more interesting papers in this volume; selecting those which, like the rich fabrics of the Indian loom, lose nothing by carriage; and leaving untouched, but by no means contemning, others that, like the produce of an Eastern garden, to be relished, must be tasted on the spot.

